# AP‐1‐Targeting Decoy Oligonucleotides: Mechanisms and Therapeutic Potential as Modulators of Inflammatory Pathways

**DOI:** 10.1111/jcmm.71178

**Published:** 2026-05-12

**Authors:** Maryam Mahjoubin‐Tehran, Samaneh Rezaei, Vasily N. Sukhorukov, Amin Jalili, Prashant Kesharwani, Amirhossein Sahebkar

**Affiliations:** ^1^ School of Pharmacy Mashhad University of Medical Sciences Mashhad Iran; ^2^ Department of Medical Biotechnology and Nanotechnology, Faculty of Medicine Mashhad University of Medical Sciences Mashhad Iran; ^3^ Institute of General Pathology and Pathophysiology Moscow Russia; ^4^ Next‐Generation Translational Nanomedicine Laboratory, Department of Pharmaceutical Sciences Dr. Harisingh Gour Vishwavidyalaya (A Central University) Sagar Madhya Pradesh India; ^5^ Biotechnology Research Center, Pharmaceutical Technology Institute Mashhad University of Medical Sciences Mashhad Iran; ^6^ Centre for Research Impact & Outcome, Chitkara College of Pharmacy Chitkara University Rajpura Punjab India; ^7^ Applied Biomedical Research Center, Basic Sciences Research Institute Mashhad University of Medical Sciences Mashhad Iran

**Keywords:** AP‐1, cancer, cardiovascular disease, decoy, oligonucleotides, transcription factors

## Abstract

Numerous cellular functions, such as apoptosis, proliferation, differentiation, survival, transformation and cell migration, are regulated by a crucial transcription factor called activator protein 1 (AP‐1). Growing evidence indicates that AP‐1 is involved in severe conditions like fibrosis, cancer, and organ damage, as well as inflammatory diseases, including rheumatoid arthritis, psoriasis, and asthma. In recent years, AP‐1 has become a significant focus in drug research. The activation of AP‐1 by TNF is crucial for essential components of the inflammatory reaction, including the expression of tissue remodelling proteases such as collagenase, as well as pro‐inflammatory cell adhesion molecules like E‐selectin. This transcription factor is formed by the assembly of jun‐jun homodimers, jun‐fos heterodimers, and jun‐ATF (Activating Transcription Factor) heterodimers. As a member of the basic leucine zipper (bZIP) class, AP‐1 regulates target genes by binding to their promoters in a sequence‐specific way. New research suggests that reducing AP‐1 function could improve various disease outcomes and treatments. Transfection of decoy oligonucleotides (ODNs) offers an innovative approach to gene therapy by targeting specific gene regulatory elements. Transcription factor decoys mimic the sites where transcription factors bind, competing with promoter regions within the cell nucleus. These molecules can regulate interactions between DNA sequences and transcription factors, which play a role in altering gene activation during both normal and disease‐related cellular processes. This review aims to summarize the effects of AP‐1‐targeted decoys on various conditions. The studies demonstrated the great promising role of AP‐1 decoys as therapeutics for various diseases, especially cardiovascular diseases and cancers. Moreover, it was shown that modifications of AP‐1 (circular and hairpin as well as phosphorothioate backbones structures) decoys made them more stable and effective.

## 1 Introduction

The nuclear transcription factor AP‐1, produced from Fos and Jun protein dimers, is associated with a wide range of cellular processes, including apoptosis, transformation, proliferation and differentiation [1]. AP‐1 frequently functions as a transcription factor that dictates cellular life‐or‐death outcomes in response to extracellular signals [2]. It is becoming more evident that the cellular environment is essential for ascertaining the role of AP‐1 in cellular outcomes.

## 2 Structure of AP‐1 and Its Dimers

**FIGURE 1:**
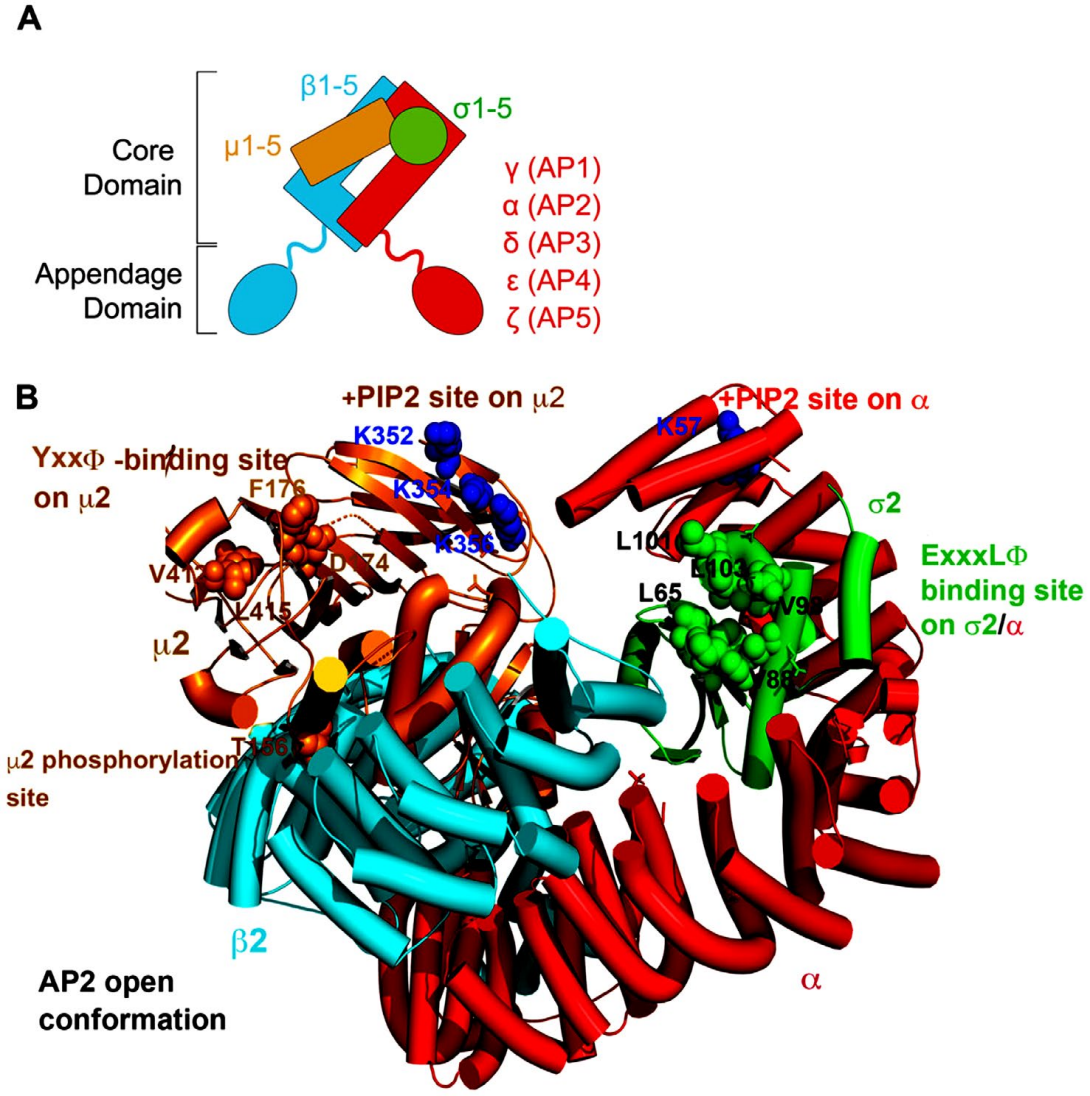
Subunit composition and an example of the structure of an AP complex. (A) A general diagram illustrating the heterotetrameric AP complex. The core or trunk domain comprises a large subunit, which is specifically designated for each complex (α, γ, δ, ε or ζ), a large β subunit, a medium μ subunit, and a small σ subunit. The appendage domains engage with various regulatory proteins, and in the instances of AP1 and AP2, with clathrin. (B) The core domain of AP2 is depicted in an ‘open’ conformation, where the sites that bind to ‘cargo proteins’—transmembrane proteins intended for specific vesicular transport—are available. The binding sites for the most prevalent and well‐characterized sorting motifs within the cytoplasmic domains of cargo proteins are illustrated: YxxΦ sequences bind to μ2, while ExxxLΦ sequences bind to a site formed by α and σ2. In both sorting motifs, Φ represents an amino acid with a bulky hydrophobic side chain, such as L, I or V. Acidic cluster sorting motifs attach to the μ subunits at basic patches (not depicted here but refer to Section 2.3.3. for further details). The phosphorylation site on μ2 (T156) is indicated; phosphorylation results in the “opening” of the complex. Additionally, the binding sites for the phospholipid PIP2 are shown, which enhances the interaction of AP2 with the inner leaflet of the plasma membrane. Tubes represent α‐helices; ribbons denote β‐strands; spheres signify binding sites. PDB code: 2XA7. This figure was adapted from the reference [3], which is licenced under a Creative Commons Attribution 4.0 International Licence.

Adaptor protein (AP) complexes are organized into five complexes: AP1‐5. While these complexes exhibit a comparable overall structure, they possess unique subunit compositions that facilitate specific functions (Figure 1). Each AP complex consists of four subunits: two large adaptins (β along with either α (AP2), γ (AP1), δ (AP3), ε (AP4) or ζ (AP5)), one medium‐sized μ subunit, and one small σ subunit [3].

**FIGURE 2:**
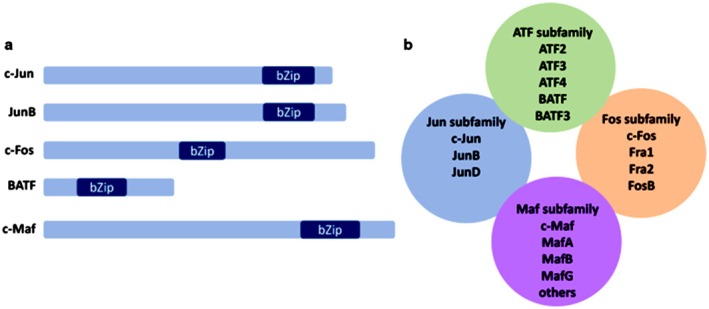
AP‐1 proteins are transcription factors containing bZip domains and are divided into four subfamilies. (a) The image shows the relative size and position of the bZip domain in typical AP‐1 proteins from different subfamilies. The dimensions of AP‐1 and the position of the bZip domain were derived from the CDD v3.18 (Conserved Domain Database) on the NCBI (National Center for Biotechnology Information) website. (b) The subfamilies ATF, Jun, Fos, and Maf within the AP‐1 family are illustrated. This Figure was adapted from the reference [2], which is licenced under a Creative Commons Attribution 4.0 International Licence.

Activator protein‐1 transcription factors (AP‐1TFs) have a basic leucine zipper (bZip) domain (Figure 2a). During the 1980s, v‐Fos and v‐Jun were identified as carcinogenic agents of FBJ‐MSV (FBJ murine osteosarcoma virus) [4] and ASV17 (avian sarcoma virus 17) [5]. Cellular homologues of c‐Jun, c‐Fos and related proteins belong to the Fos and Jun subfamilies [2]. Several members of the Maf and ATF subfamilies have been integrated into the AP‐1 family to expand its functions (Figure 2b).

**FIGURE 3:**
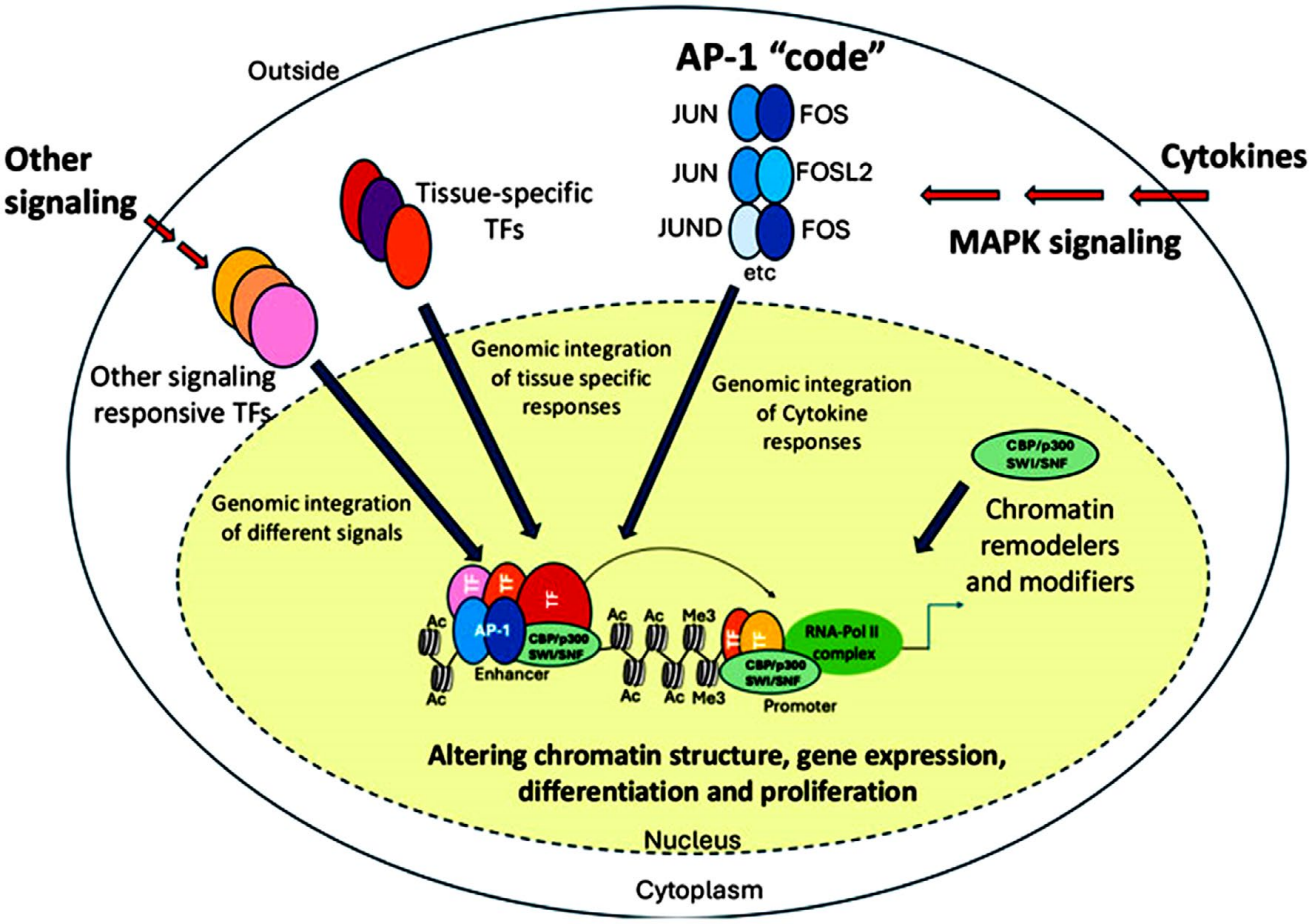
The influence of AP‐1 on gene expression. A model illustrating how various combinations of AP‐1 family members collaborate with chromatin remodelers/modifiers, along with tissue‐specific and other signalling‐dependent transcription factors (TFs), to enhance gene expression in response to external signals and developmental cues. AP‐1 dimers, chromatin modifiers/remodelers, and other classes of TFs are represented by distinct shapes and colours. Modifications to histone tails, facilitated by recruited chromatin modifiers, are also depicted. This figure was adapted from the reference [6], which is licenced under the Creative Commons Attribution 4.0 International Licence.

The AP‐1 transcription factor (TF) consists of homo‐ or heterodimers formed from proteins of the jun‐atf, jun‐fos or jun‐jun families. AP‐1 is classified as a bZip TF. It activates or represses target gene promoters by binding in a sequence‐specific manner. AP‐1 proteins regulate processes such as cell proliferation, migration, apoptosis, differentiation, survival, growth and transformation [2]. The composition of the dimers, the amount of each dimerization partner, post‐translational modifications, and interactions with accessory proteins determine whether an AP‐1 factor regulates a target gene (Figure 3) [2].

The AP‐1 proteins function as dimers, which can be either homodimers or heterodimers [7]. Leucine zippers facilitate dimerization. In this domain, leucine side chains interact with the alpha helix, enabling the leucine zipper to form dimers [8, 9]. The core domain is essential for engaging with DNA [8]. AP‐1 proteins bind to cAMP‐responsive elements (CRE) (TGACGTCA), TPA‐responsive elements (TRE) (TGA(G/C)TCA), and similar sequences [10, 11, 12, 13]. Dimers differ in DNA binding and transcription activity. C‐Fos:c‐Jun dimers prefer TRE regions, while ATF dimers target CRE regions. Furthermore, heterodimers (C‐Fos:c‐Jun) exhibit a higher affinity for TRE than homodimers (c‐Jun: c‐Jun), and dimers with B‐Jun tend to be less active in transcription [14, 15]. These proteins are thought to be transcriptional activators, although they may also act as repressors. Therefore, the AP‐1 family forms various dimers with distinct transcriptional and DNA‐binding roles.

The complexities of the issue originate with AP‐1 itself. AP‐1 is not a single protein; rather, it is a conglomerate of dimers formed from the Jun family members (JunB, c‐Jun and JunD) of basic leucine‐zipper (bZIP) proteins, in conjunction with related proteins from the Fos (FosB, c‐Fos, Fra1 and Fra2) as well as ATF families. These proteins exhibit distinct expression and regulation, resulting in a complex combination of AP‐1 dimers inside every cell, each having nuanced activities. The result of AP‐1 activation relies on the intricate combination of AP‐1 dimers. The AP‐1 complex interacts with a palindromic DNA motif to modulate gene expression. AP‐1 activity is stimulated by a very wide variety of extracellular stimuli, involving hormones, mitogens, extracellular matrix components, and genotoxic chemicals. Numerous stimuli activate c‐Jun N‐terminal kinases (JNKs), resulting in the Jun proteins' phosphorylation and increased transcriptional activity. Elevations in Jun and Fos protein levels, together with JNK activity, have been shown in several instances of cellular apoptosis [16].

AP‐1 proteins associate with cAMP‐responsive elements (CRE) (TGACGTCA), 12‐O‐tetradecanoylphorbol‐13‐acetate (TPA) responsive elements (TRE) (TGA(G/C)TCA), and similar sequences. Distinct dimers exhibit variability in their transcriptional functions and DNA‐binding features. For instance, c‐Jun:c‐Fos dimers exhibit a preference for TRE sites, while c‐Jun:ATF dimers exhibit a preference for CRE sites [16]. Furthermore, c‐Jun:c‐Fos heterodimers possess a greater affinity for TRE sites compared to c‐Jun:c‐Jun homodimers, whereas dimers including JunB show reduced transcriptional activity relative to those containing c‐Jun [13]. While these proteins are mainly assumed to serve as transcriptional activators, there are cases when they seem to behave as repressors. The AP‐1 family comprises a wide range of proteins that form a wide variety of dimers with distinct DNA binding and transcriptional functions [12]. AP‐1 family proteins, unsurprisingly, govern an extensive variety of cellular and biological functions. This encompasses apoptosis, the cell cycle, cellular proliferation, autophagy, and lipid synthesis. Additionally, AP‐1 proteins control migration and invasion by modulating the cytoskeleton and are associated with inflammatory illnesses, bone growth, the neurological system, immune cell formation and activation, as well as cancer [2].

## 3 Functions of AP‐1

**FIGURE 4:**
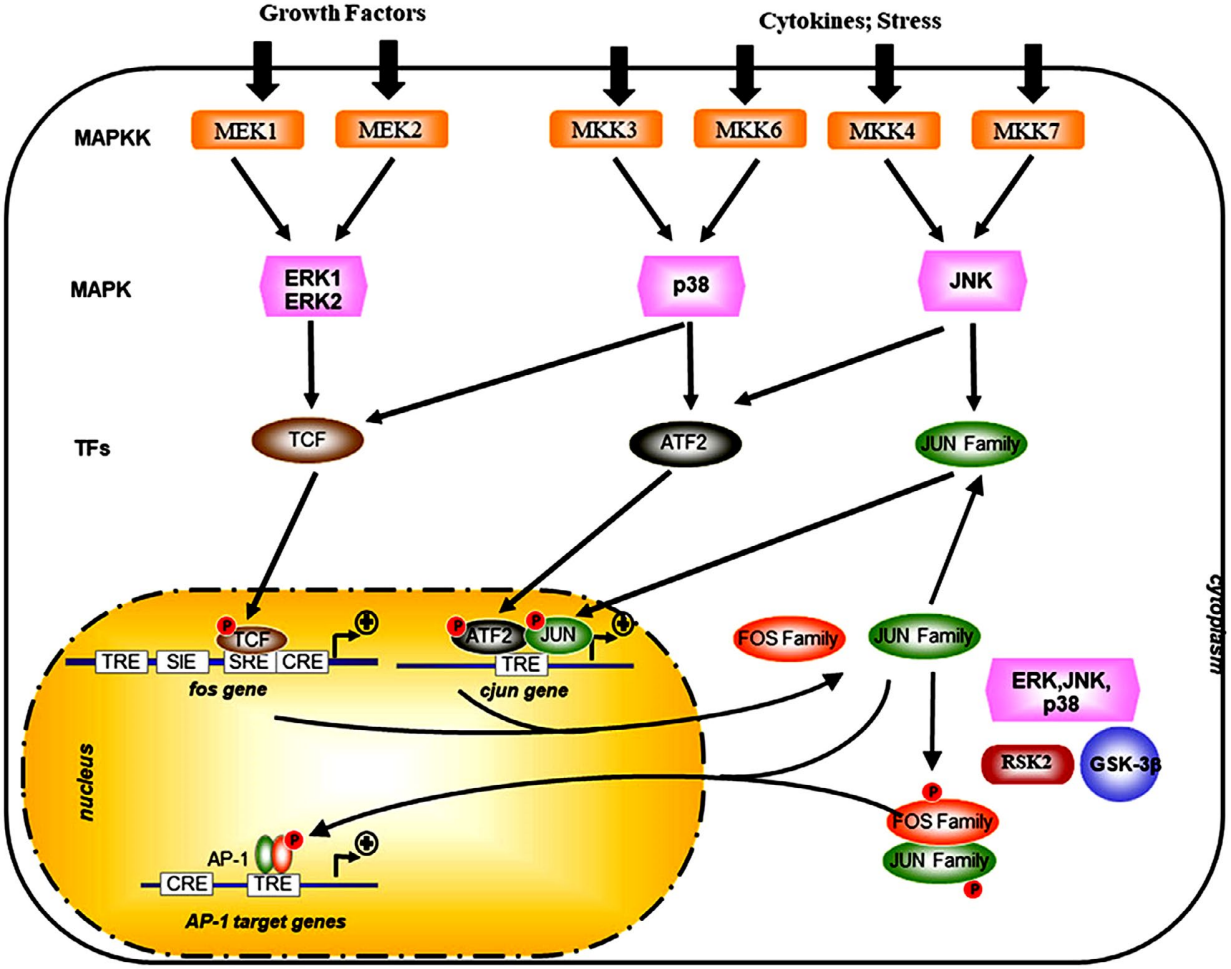
Transcriptional and post‐translational activation of AP‐1. The activity of AP‐1 is enhanced by external factors such as growth factors or inflammatory cytokines, along with a complex network of kinases, including the mitogen‐activated protein kinases (MAPKs) from the extracellular‐signal‐regulated kinase (ERK), p38, and JUN amino‐terminal kinase (JNK) families. The regulation of AP‐1 activity, which encompasses its transactivating ability, DNA‐binding capacity, and the stability of its components, is influenced by posttranslational phosphorylation by various kinases. CRE, CAMP‐response element; GSK‐3β, Glycogen synthase kinase‐3β; MAPKK, MAPK kinase; RSK2, Ribosomal S6 kinase 2; SIE, Sis‐inducible element; SRE, Serum‐response element; TCF, Ternary complex factor; TRE, TPA‐responsive element. This figure was adapted from the reference [23].

Naturally, the AP‐1 family regulates many cellular and biological processes, including cell cycle and proliferation [17, 18], apoptosis [16, 17, 18], autophagy [19] and lipid production [20]. Additionally, AP‐1 proteins influence invasion and migration by regulating the cytoskeleton and are linked to inflammatory disorders, bone growth, nervous system function, immune development and activation, and cancer [2]. Gene expression in diverse biological conditions is initiated and regulated at the transcriptional level through interactions between transcription factors and promoter regions containing specific DNA elements. AP‐1 binds to particular DNA sequences present in many genes that control various cellular processes through transcriptional and post‐translational activation (Figure 4) [21]. Recent research indicates that AP‐1 is a key transcription factor that increases the expression of genes linked to various diseases [22].

Recent transcriptome studies have shown the pivotal functions of AP‐1 in cellular processes and many illnesses [24, 25, 26, 27, 28]. Furthermore, genome‐wide research revealed extensive chromatin regulatory pathways for AP‐1, including pioneer function. Pioneer factors (PFs) are often regarded as a distinct category of sequence‐specific transcription factors (TFs) capable of recognizing nucleosome‐embedded patterns, infiltrating dense chromatin, and creating open chromatin areas [29]. Pioneer factors are a category of transcription factors that play crucial functions in gene regulation throughout development. Their distinctive capacity to attach to compacted chromatin, facilitating its remodelling for gene expression, distinguishes them from other regulatory proteins and renders them crucial participants in cellular differentiation, destiny determination, and reprogramming [30]. Pioneer factors, a kind of transcription factor, were originally identified during studies on the formation of enhancer regulatory complexes in silent chromatin throughout embryonic development, which subsequently activates a tissue‐specific gene network. Pioneer transcription factors were later discovered to facilitate hormone‐responsive gene networks in human malignancies and to intentionally alter cellular destiny in culture [31].

Transcription factors may attach to these histone‐modifying enzymes in a variety of ways. They can also function as pioneer factors by binding to nucleosomes and directly opening chromatin [31]. A recent study by Larsen et al. found that heterodimeric partners of the AP‐1 family, including JUN, subsequently access, modify chromatin, and trigger transcription [32].

## 4 AP‐1 as a Modulator of Inflammatory Pathways

AP‐1 operates under pathophysiological circumstances, including inflammation. Inflammatory cytokines from the TNF family orchestrate diverse cellular and organismal inflammatory responses and play a crucial role in the pathogenesis of several significant diseases, including septic shock, arthritis, inflammatory bowel disease, and potentially type II diabetes [33]. A significant portion of the responses to these cytokines demand de novo gene expression facilitated by the AP‐1 heterodimeric transcription factor [33]. The activation of AP‐1 entails the direct dephosphorylation and phosphorylation of its components, with the resulting activation and phosphorylation of transcription factors that enhance the production of c‐jun or c‐fos. These events may be individually triggered by several signalling pathways [1]. The activation of AP‐1 by TNF is crucial for essential components of the inflammatory response, including the development of pro‐inflammatory cell adhesion molecules like E‐selectin and the expression of tissue remodelling proteases such as collagenase [1].

## 5 Frontline Treatments Directed at AP‐1

Numerous medicines target AP‐1 transcriptional elements. For instance, the small molecule tyrosine kinase inhibitor Imatinib targets PDGFRβ, obstructing antibodies that disrupt the PD‐1/PD‐L1 interaction (Pembrolizumab, Nivolumab), a bispecific antibody that recruits NK cells to CD30‐expressing cells (AFM13), and an antibody‐drug conjugate that targets CD30‐positive cells (Brentuximab Vedotin). Imatinib's inhibition of PDGFRβ kinase activity effectively treated an ALK+ ALCL patient who was unresponsive to treatment and recurred after autologous stem cell transplantation. Furthermore, other medicines aimed against CD30 or the PD‐1/PD‐L1 interaction have been developed, which will be further discussed upon in the subsequent sub‐section [2].

Proteomic and microarray analyses have been conducted to uncover genome‐wide dysregulated genes in knockdown cells for some family members. Expanding these tests to include more AP‐1 proteins, albeit technically more demanding, will provide a more thorough comprehension of the cellular processes governed by these transcription factors when several AP‐1 proteins are knocked down or wiped out [34]. Similarly, chromatin immunoprecipitation‐sequencing (ChIP‐Seq) techniques will enhance these studies by comprehensively defining genomic loci occupied by these transcription factors and finding genes that are more likely to be direct transcriptional targets. Furthermore, ChIP‐Seq studies will elucidate which genes are predominantly controlled by specific AP‐1 proteins as opposed to those influenced by numerous family members. Similarly, whereas several AP‐1 proteins are aberrantly produced in these lymphomas, there is less knowledge on the prevalence and function of certain AP‐1 dimers. Quantitative mass spectrometry investigations and research using specific AP‐1 dimers will elucidate these inquiries [34]. As more AP‐1‐regulated genes are discovered, certain genes or the signalling pathways they are associated with may emerge as innovative therapeutic options. The AP‐1 family proteins may serve as potential therapeutic targets [35, 36]. Oligodeoxynucleotide decoys that disrupt AP‐1 DNA binding are now under investigation as potential treatments.

## 6 Decoy ODNs: Promising Therapeutic Approaches

Small double‐stranded DNA (dsDNA) molecules, known as decoy oligodeoxynucleotides (ODNs), contain a specific transcription factor (TF) binding sequence within a gene's promoter. The alignment of multiple target gene promoter binding sites shows that this sequence is conserved across different species [37].

Decoy oligonucleotides have become a valuable tool in a new class of antigen therapies introduced in recent years. The technology behind decoy oligonucleotides has attracted significant interest for treating and potentially curing various diseases and abnormal physiological conditions, as they provide a logical approach to designing and selectively regulating specific gene expression [37]. Decoy oligonucleotides are widely used as inhibitors of specific gene expression because they offer an exciting opportunity to activate or suppress a particular gene without affecting the functions of other genes. Recent advances in decoy oligonucleotides are quickly leading to new insights into the causes and treatments of inflammatory conditions, cancer, and other immune disorders [38, 39, 40].

To minimize non‐specific off‐target effects and enhance the stability of decoy ODNs, researchers used numerous methodologies. Crinelli et al. have examined the use of conformationally constrained nucleotides in the development of decoy molecules for NF‐κB. Beginning with a synthetic double‐stranded oligonucleotide featuring the κB consensus binding region, scientists produced a series of decoy molecules modified to varying degrees and at different places using locked nucleic acids (LNAs). Furthermore, they said that including terminal LNA bases, external to the κB sequence, to create LNA–DNA–LNA co‐polymers was enough to provide significant protection against nuclease degradation, without hindering transcription factor binding [41]. Additionally, chimeric decoy oligodeoxynucleotides containing consensus sequences for two transcription factors were employed to suppress multiple transcription factors simultaneously [42, 43]. Addition of the hairpin sequence to ODN has been shown to confer stability and nuclease resistance [44]. Gwon designed ring‐type decoy ODN which includes two consensus sequences for binding and trapping two transcription factors (HIF‐1α and STAT5) [42].

This study reports the progress in developing decoy oligonucleotides targeting AP‐1 as a promising approach within a new category of molecular therapy for AP‐1 inhibition (Table 1).

**TABLE 1:** Therapeutic effects of AP‐1 decoy ODN.

Decoy name	Disease	Modifications	Delivery	Cell line/model	Key findings	References
AP‐1 decoy ODN	Vascular remodelling or hypertension‐related vascular disease involving abnormal vascular smooth muscle growth	Ds ODN designed to mimic AP‐1 binding site; often phosphorothioate backbone for stability	Transfection into cultured VSMC	VSMC cultured from rat models such as Wistar‐Kyoto (WKY) rats and Sprague–Dawley rats	AP‐1 decoy ODN blocks Ang II‐stimulated AP‐1 activity, leading to inhibition of TGF‐beta gene expression and reduction in VSMC growth	[45]
CRE‐TF decoy ODN	Tumour	Phosphorothioate backbone	Cell penetration	Tumour cell lines	Inhibition of tumour cell proliferation; specific interference with CRE‐ and AP‐1‐directed transcription	[46]
AP‐1 ODN	Cardiovascular disease (vascular remodelling)	Phosphorothioate backbone for stability	Transfection into cultured human VSMCs	Cultured human VSMC	PAI‐1 gene expression inhibition via Ang II and high glucose	[47]
Circular dumbbell AP‐1 decoy ODN (CDODN) and phosphorothioate linear decoy ODN (PSODN)	Atherosclerosis, restenosis after angioplasty	Circular dumbbell structure for CDODN; phosphorothioate backbone for PSODN	HVJ‐liposome method in vivo; transfection in vitro	Rat VSMCs, carotid artery balloon injury model	Strong inhibition of VSMC migration and proliferation; inhibition of neointima generation following vascular injury; CDODN is more stable and effective than PSODN	[48]
AP‐1 ODN	Human squamous cell carcinoma	Phosphorothioate backbone	Transfection into cultured carcinoma cells	Human squamous cell carcinoma cell lines	Inhibition of EGF‐related invasion	[49]
AP‐1 ODN	Diabetic nephropathy	Phosphorothioate backbone	HVJ‐liposome method in vivo; transfection in vitro	Cultured mesangial cells; streptozotocin‐induced diabetic rat model	Inhibition of Ang II‐high glucose‐related mesangial cell proopagation and ECM production; reduced TGF‐β1 and PAI‐1 expression in vivo	[22]
AP‐1 TF decoy ODN	Keloid fibroblasts (keloid scars)	Phosphorothioate backbone	Transfection into cultured keloid fibroblasts	Cultured human keloid fibroblasts	Suppression of TGF‐β‐related type I collagen gene expression	[50]
AP‐1 ODN	Pulmonary fibrosis (fibrotic lung disease)	Phosphorothioate backbone	Transfection into cultured pulmonary fibroblasts	Cultured pulmonary fibroblasts; bleomycin‐A5‐induced fibrosis model	Modulation of MMP‐2/TIMP‐1 imbalance; inhibition of fibrotic responses induced by bleomycin‐A5	[51]
AP‐1 TF decoy ODN	Scleroderma (systemic sclerosis)	Phosphorothioate backbone	Transfection into cultured scleroderma fibroblasts	Cultured human scleroderma fibroblasts	Decreasing TGF‐β‐ralated type I collagen synthesis	[52]
Circular dumbbell AP‐1 ODN (CDODN) and phosphorothioate linear decoy ODN (PSODN)	Intimal hyperplasia, vein graft restenosis	Circular dumbbell structure for CDODN; phosphorothioate backbone for PSODN	HVJ‐liposome method in vivo	Autogenous vein grafts in mongrel dogs	Marked inhibition of intimal hyperplasia in autogenous vein grafts; CDODN more stable and effective than PSODN	[53]
AP‐1 decoy ODN and STAT‐1	Acute rejection in cardiac transplantation	Phosphorothioate backbone for stability	In vivo delivery via repetitive administration in rat cardiac allograft model	Rat cardiac allograft transplantation model	Delay in acute rejection; prolonged cardiac allograft survival; suppression of inflammatory gene expression	[54]
Thioaptamer decoy targeting AP‐1 proteins	Arenavirus infections	Thioaptamer backbone (sulphur‐modified nucleotides)	Not explicitly detailed; likely in vitro and in vivo via transfection or injection	Cell cultures and animal infection models relevant to arenavirus	Modulated cytokine expression; influenced infection outcomes by impacting AP‐1‐mediated pathways	[55]
AP‐1 TF decoy ODN	Scleroderma (systemic sclerosis)	Phosphorothioate backbone	Transfection into cultured scleroderma fibroblasts	Cultured human scleroderma fibroblasts	Reduction of TGF‐beta1 induced cell growth by inhibiting cyclin E expression	[21]
AP‐1 ODN	Experimental colitis (intestinal inflammation) in mice	Phosphorothioate backbone	Intraperitoneal administration or appropriate in vivo delivery in mice	Murine model of experimental colitis	Significant attenuation of intestinal inflammation and inflammatory cytokine expression	[56]
AP‐1 and STAT‐1 ODN	Transplant vasculopathy, cardiac allograft rejection	Phosphorothioate backbone	In vivo delivery to rat cardiac allografts	Rat cardiac allograft transplantation model	Attenuation of transplant vasculopathy; reduced inflammatory response and graft rejection	[57]
AP‐1 ODN	Cardiac fibrosis, fibroblast proliferation	Phosphorothioate backbone	Transfection into cultured neonatal rat cardiac fibroblasts	Neonatal rat cardiac fibroblasts	Inhibition of fibroblast proliferation and collagen I & III synthesis	[58]
AP‐1 ODN	Oxidative stress‐induced cardiac fibroblast proliferation and ECM remodelling	Phosphorothioate backbone	Transfection into cultured rat cardiac fibroblasts	Neonatal rat cardiac fibroblasts	Strong inhibition of oxidative stress (XXO)‐induced fibroblast proliferation and MMP gene expression	[59]
SMAD ODN and Dual AP‐1	Tissue scarring and fibrosis	Phosphorothioate backbone	In vivo administration in mice	Mouse model of tissue fibrosis	Suppression of tissue fibrosis and scar formation; reduction in fibrotic gene expression	[60]
AP‐1 consensus dODN	Marfan Syndrome	Phosphorothioate	Naked ex vivo	mgR/mgR mice, mAoSMCs, HUASMC	Lowering MMP expression/activity, macrophage infiltration, and elastolysis	[61]
Hairpin AP‐1 consensus dODN	Marfan Syndrome	Phosphorothioate	Naked ex vivo	mgR/mgR mice, mAoSMCs	Reduced migration, ROS formation, and MMP expression/activity	[61]
Hairpin AP‐1 ODN	Transplant vasculopathy (TV)	Single‐stranded, self‐complementary phosphorothioate‐modified hairpin DNA decoy ODN; higher thermal stability and exonuclease resistance	Incubation of aortic allografts with dODN ex vivo during transplantation (intraoperative treatment)	Mouse heterotopic aortic allograft model: DBA/2 donor to C57BL/6 recipient mice	Significant reduction in intima‐to‐media (I/M) ratio, decreased SMC α‐Actin expression, neointima formation reduced, reduced macrophage infiltration, reduced VCAM‐1 expression, no significant effect on MMPs or fibrosis marker CTGF at 30 days post‐transplant	[62]
RNA hairpin AP‐1 dON	Transplant vasculopathy	RNA hairpin, targeting peptide in AAV9‐SLR capsid	AAV‐mediated, ex vivo transduction	Mouse aortic allografts	Reduced intima‐to‐media ratio, decreased adhesion molecules, cytokines, MMP‐9‐positive cells, inflammatory cell infiltration, proliferative SMCs	[63]
RNA hairpin AP‐1 decoy ODN (hp dON)	Marfan syndrome	RNA hairpin decoy ODN neutralizing AP‐1 TF; stable expression	AAV vector ex vivo transduction of aortic grafts implanted in mice	Fibrillin‐1 hypomorphic (mgR/mgR) mice, primary aortic smooth muscle cells	Reduced MMP expression/activity, monocyte infiltration, ROS formation, and maintained elastin integrity	[63]

Preclinical research using relevant animal models has effectively evaluated TF decoy (TFD) ODNs. Initial clinical studies can examine the safety and effectiveness of these ODNs. This suggests that the neutralizing TF recognizes disease‐related genes consistently across different species [64].

Most pharmaceutical ODNs have phosphorothioate links, which degrade less than phosphate ester interactions in vivo due to the action of multiple extracellular and intracellular nucleases [65]. This chemical modification was initially employed in antisense technology because it enhances the stability of single‐stranded ODNs and has also been shown to improve cellular uptake and the bioavailability of antisense ODNs in vivo [66]. Furthermore, conformationally constrained nucleotides have been incorporated into the design of TFD molecules. The resulting ODNs are often modified to varying degrees and at different sites with locked nucleic acids (LNAs). These nucleic acids are modified RNA nucleotides characterized by an additional bridge connecting the 2′‐oxygen atom to the 4′‐carbon atom. Adding terminal LNA bases outside the target TF binding area protects against nuclease digestion without affecting TF binding [41]. Recent studies have reported various chemical modifications that improve the thermal stability and nuclease resistance of these ODNs [67]. One promising approach involves using hairpin‐shaped dsODNs or dumbbell‐shaped (ribbon‐type decoy ODNs). Circular ribbon‐type TFD ODNs have notable exonuclease resistance and thermal stability because they lack terminal nucleotide residues [68] and are suitable for systemic delivery [69]. Phosphorothioate modifications in specific regions of ribbon‐type TFD ODNs further enhance their endonuclease resistance for systemic administration [69]. Chemical cross‐linking, which employs alkynyl‐NaNO nucleosides and a stilbene linker, has been used to address the challenge of producing large quantities of ribbon‐type decoy ODNs via enzymatic methods [66]. A synthetic, hairpin‐shaped DNA ODN with an integrated single diphosphoryl disulfide linkage and a consensus NF‐κB‐binding region was shown to covalently bind to the p50 subunit of NF‐κB [70].

## 7 Decoy Oligonucleotides Targeting AP‐1

Morishita et al. transfected AP‐1 decoy ODN into VSMCs from WKY rats, which provided latent but inactive TGF‐beta, and Sprague–Dawley rats, which provided both active and latent TGF‐beta upon stimulation with Ang II. AP‐1, but not mismatched decoy ODN, abolished Ang II‐stimulated TGF‐beta gene expression and synthesis in both VSMCs. In WKY rats, the AP‐1 decoy did not affect DNA, RNA, or protein production. Ang II activation by AP‐1 decoy ODN in Sprague–Dawley cells increased cell number without changing RNA or protein levels. These data demonstrate that Ang II enhances AP‐1 complex–mediated TGF‐beta production. Ang II‐stimulated AP‐1 complexes may limit cell proliferation in Sprague–Dawley VSMCs. They showed that the decoy strategy could be used to explore the unique role of cis elements in endogenous gene control, such as Ang II‐associated TGF‐beta production [45].

The suppression of specific transcriptional regulatory proteins to modify gene transcription offers considerable therapeutic potential. In cells, decoy cis‐elements composed of synthetic double‐stranded phosphorothioate ODNs with high specificity for target transcription factors can influence gene expression. The CRE‐TF complex is a versatile activator involved in activating numerous viral and cellular genes. The palindromic CRE cis‐element, TGACGTCA, allows a synthetic single‐stranded ODN containing the CRE sequence to self‐hybridize, forming either a duplex or hairpin structure. Researchers observed that CRE‐palindromic ODNs can enter cells, compete with CRE enhancers for transcription factors, and selectively inhibit AP‐1 and CRE‐mediated transcription in animal models. These ODNs prevented tumour cell growth while sparing noncancerous cells. This decoy ODN (dON) approach holds significant promise for understanding cellular regulation mechanisms and treating cancer and other diseases [46].

Hyperglycemia and Ang II upregulate the PAI‐1 gene in human VSMC. Ahn et al. tested the hypothesis that hyperglycemia and Ang II promote PAI‐1 gene expression through AP‐1 binding sites. Using a ds‐cis‐element AP‐1 decoy ODN, they investigated AP‐1's role in PAI‐1 gene expression in VSMCs stimulated with high D‐glucose and Ang II. The AP‐1 decoy ODN competed for activator protein‐1 activity, but not a mismatched one, after high glucose and Ang II treatment. The AP‐1 decoy ODN significantly decreased PAI‐1 expression, while high glucose and Ang II increased it. PAI‐1 promoter studies indicated that hyperglycemia and Ang II activity on AP‐1 sites enhance PAI‐1 expression. High glucose and co‐stimulation involving elevated glucose and Ang II activation of AP‐1 were inhibited entirely by calchostin C (and to some extent by genistein). Ang II and hyperglycemia promote PAI‐1 expression via AP‐1 binding regions. PKC activation is essential for signal transduction when high glucose and Ang II activate AP‐1. These findings suggest that AP‐1 is vital for PAI‐1 expression [47].

Ahn et al. developed a novel CDODN therapy which targets AP‐1 for restenosis following angioplasty. This CDODN was more resistant to degradation than PSODN and maintained its structure after exposure to serum or exonuclease III. Transfection of AP‐1 decoy ODNs significantly reduced VSMC migration, proliferation, and serum and glucose‐induced PCNA and cyclin A gene expression. Using a balloon injury model of the rat carotid artery, HVJ‐liposome‐delivered AP‐1 decoy ODNs significantly reduced neointimal growth. CDODN suppressed VSMC proliferation and neointimal development more effectively than PSODN in both in vivo and in vitro studies. Their results demonstrated that VSMC growth following vascular injury depends on AP‐1 activation. Stable CDODN targeting AP‐1 activity combined with the highly effective HVJ‐liposome technique offers a new treatment option for angioplasty restenosis in humans [48].

The extracellular protease cascade in squamous cell carcinoma (SCC) cells breaks down the extracellular matrix (ECM) through plasmin, uPA, and the collagenase MMP family. Shiratsuchi et al. discovered that oral SCC cells invaded Matrigel after treatment with EGF. The synthesized inhibitors and uPA antibodies reduced EGF‐related cell invasion. EGF also increased TF AP‐1‐DNA binding and the expression of uPAR/uPA proteins and mRNA. Dexamethasone prevented these EGF‐induced changes and also increased PAI‐1 (plasminogen activator inhibitor type 1). Transfecting SCC cells with AP‐1 decoy ODNs inhibited EGF‐induced uPA and uPAR production and invasion of Matrigel. The transcription factor AP‐1 mediates the uPA‐plasmin cascade used by oral SCC cells to invade Matrigel. The uPA‐uPAR interaction enhances proteolytic activity and uPAR‐related signalling, promoting motility and invasion. DEX suppresses uPA and uPAR, making it a potential therapy for oral SCC [49].

Diabetic nephropathy causes glomerular mesangium growth due to cell proliferation and ECM protein buildup, leading to glomerulosclerosis and kidney failure. The TF AP‐1 regulates genes involved in cell proliferation and ECM production. Ahn et al. created a decoy ODN‐based treatment to explore whether AP‐1 influences ECM gene expression. In mesangial cell cultures, AP‐1 decoy ODN markedly decreased cell proliferation induced by angiotensin II and high glucose levels, and it also reduced ECM gene expression. The AP‐1 decoy ODN delivered via HVJ‐liposomes nearly eliminated TGF‐beta1 and PAI‐1 expression in streptozotocin‐induced diabetic rat kidneys. They observed that mesangial cell proliferation and ECM production depend on AP‐1 activation during high hyperglycemia and the presence of angiotensin II. The combination of stable AP‐1 decoy ODN and effective HVJ‐liposome delivery offers a groundbreaking molecular therapy for preventing diabetic nephropathy [22].

Keloids recur due to excessive collagen deposition even after removal, and this condition has no recognized cause. TGF‐β1 promotes excessive ECM deposition, mainly type I collagen, leading to fibrosis and keloid formation. Abnormal AP‐1 activity in response to TGF‐β stimulation may cause overexpression of type I collagen. Kim et al. studied how decoy ODN targeting the type I collagen gene affects TGF‐β1‐treated keloid fibroblasts and examined the role of AP‐1 as a transcription factor. Compared to TGF‐β1 treatment alone, AP‐1 decoy ODN therapy reduced AP‐1 binding activity by 0.4‐fold in keloid fibroblasts. The decoy treatment also decreased TGF‐β1‐induced α1(I) procollagen mRNA by 76% in northern blot analysis. Injection of the decoy reduced TGF‐β1‐induced α2(I) procollagen promoter activity by about 51%. Keloid fibroblasts treated with TGF‐β1 and the decoy showed less collagen immunosignal than those treated with TGF‐β alone. These findings suggest that TGF‐β1‐related type I collagen transcription in keloid cells depends on AP‐1 activation. Furthermore, AP‐1 decoy ODN decreases type I collagen gene expression in vitro in TGF‐β1‐associated keloid cells, which may help limit keloid development in vivo [50].

Ma et al. examined how AP‐1 decoy affected the imbalance of MMP‐2 and TIMP‐1 in lung fibroblasts caused by BLM‐A5. Before exposure to BLM‐A5, pulmonary fibroblasts were cultured and treated using AP‐1 Decoy. Gelatin zymography measured MMP activity in the medium. ELISA was used to measure TIMP‐1 protein levels in the medium. RT‐PCR was employed to assess MMP‐2 and TIMP‐1 mRNA expression. BLM‐A5 increased MMP‐2 activity at 12 h, but AP‐1 Decoy reduced it. After 12 and 24 h, BLM‐A5 elevated TIMP‐1 protein, mRNA, and the absorbance ratio to beta‐actin, while AP‐1 Decoy diminished this upregulation. Unlike the control group, the AP‐1 Decoy group showed no significant differences in these measures. The mutant AP‐1 Decoy had different effects on MMP‐2 and TIMP‐1 expression in pulmonary fibroblasts. Overall, AP‐1 Decoy suppresses BLM‐A5‐induced upregulation of MMP‐2 and TIMP‐1 in pulmonary fibroblasts [51].

Cho et al. examined circular dumbbell AP‐1 decoy ODN (CDODN) versus a mismatched AP‐1 decoy ODN (MODN) as a control using normal and scleroderma fibroblasts obtained from leftover cosmetic surgery tissue. Before TGF‐β1 treatment, both cells were transfected with CDODN and MODN for 24 h. To study how AP‐1 decoy ODN influences TGF‐β1‐associated AP‐1 binding, scleroderma fibroblasts received MODN or CDODN before treatment. CDODN transfection significantly inhibited AP‐1 binding activity, whereas MODN did not. Transfection with CDODN markedly decreased AP‐1 binding in TGF‐β1‐positive cells but not in MODN‐positive cells. They found that CDODN transfection significantly reduced type I collagen promoter activity in TGF‐β1‐treated cells and scleroderma fibroblasts, indicating gene downregulation [52].

The most common cause of late vein graft failure is intimal hyperplasia. The primary processes involve smooth muscle cell migration and proliferation. Pharmacological strategies aimed at improving vein graft patency have shown limited success. Cho et al. evaluated the effect of the AP‐1 decoy on intimal hyperplasia using an animal model. The jugular vein was transfected with HVJ‐liposomes containing either the AP‐1 decoy or scrambled ODNs. A pretreated jugular vein was used as an interposition graft between the transected femoral arteries. The graft was collected 16 weeks after the procedure. Analysis of the intimal area showed significantly lower values in the AP‐1 decoy‐treated group compared to the control. AP‐1 decoy with HVJ‐liposomes effectively prevented intimal hyperplasia associated with an autogenous vein graft in mongrel dogs [53].

Cell‐mediated acute cardiac rejection increases leukocyte infiltration into the transplanted myocardium. STAT‐1 and AP‐1 control the production of vascular adhesion molecules. Hölschermann et al. studied acute cardiac allograft rejection in rats after decoy ODN therapy targeting TFs AP‐1 and STAT‐1. After administering STAT‐1 or AP‐1 decoy ODNs or mutant control ODNs, Lewis (LEW) rats received rat cardiac allografts. Treatment with these decoy ODNs improved the survival of the cardiac transplants. Immunohistochemical analysis on days 1, 3 and 6 showed that leukocyte infiltration, primarily T‐cells, was significantly reduced in decoy ODN‐treated grafts by day 3. Additionally, immunohistochemistry revealed that decoy ODN‐treated grafts had notably lower expression of endothelial VCAM‐1 and ICAM‐1. By decreasing endothelial adhesion molecules and infiltration, therapies using AP‐1 and STAT‐1 decoy ODNs delay acute rejection. A new application of decoy ODNs in cardioplegic solutions could protect transplanted organs from initial injury, preserve organ function, and bridge the critical period after transplantation when immunosuppressive drugs are ineffective [54].

A variety of viruses, especially arenaviruses, can cause viral hemorrhagic fever (VHF). It is believed that dysregulation of cytokine synthesis plays a role in its development. Both Lassa and Pichinde viruses tend to infect macrophages and reticuloendothelial cells, and they appear to inhibit the typical macrophage response to viral infections. XBY‐S2, a decoy thioaptamer, was designed and shown to bind the AP‐1 protein. It was demonstrated that XBY‐S2 interacts with JunB and Fra‐2, leading to increased production of TNF‐alpha, IL‐6 and IL‐8, while reducing attachment to AP‐1 promoter elements. Injecting XBY‐S2 into guinea pigs infected with Pichinde virus reduced virus‐related mortality and boosted cytokine production from primary guinea pig macrophages, potentially improving survival rates in these infected animals. The study shows that thioaptamers may influence the outcomes of in vivo viral infections caused by arenaviruses by modulating transcription factors that control the immune response [71].

In scleroderma, the TGF‐β signalling pathway is essential for forming type I and III collagen. AP‐1 plays a key role in regulating type I collagen production, which is triggered by TGF‐β1. However, AP‐1's role in scleroderma fibroblast proliferation remains unclear. The researcher examined the effect of AP‐1 decoy ODN on the growth of TGF‐β1‐treated scleroderma fibroblasts. The study explored how AP‐1 decoy ODN influences cell cycle proteins to promote fibroblast growth—introducing AP‐1 decoy ODN into scleroderma fibroblasts reduced growth by decreasing cyclin E levels. These findings suggest that AP‐1 decoy ODN may slow fibroblast growth in scleroderma, indicating its potential as a treatment [55].

Moriyama et al. tested a decoy ODN targeting AP‐1 to inhibit DSS (dextran sulphate sodium)‐induced colitis in an animal model. Their functional efficiency was evaluated in vitro using a luciferase reporter gene assay and flagellin‐induced IL‐8 production in HCT‐15 cells treated with scrambled ODNs and AP‐1 decoy. Mice received intraperitoneal injections of either scrambled ODNs or decoy. The AP‐1 decoy ODN significantly reduced colon shrinkage, DSS‐induced weight loss, and histological damage, while scrambled ODNs had no effect in mice. Additionally, the AP‐1 decoy ODN lowered levels of myeloperoxidase, IL‐1β, and TNF‐α in colonic tissue. Both the NF‐kB decoy ODN and the AP‐1 decoy decreased intestinal inflammation. The double‐strand decoy ODN targeting AP‐1 reduced intestinal responses associated with experimental colitis, supporting the idea that targeting pro‐inflammatory TFs could be a promising therapy for IBD [21].

Previous studies have shown that ex vivo donor allograft therapy with ODNs targeting TFs like STAT‐1 or AP‐1 may extend cardiac allograft survival and delay acute rejection. Stadlbauer et al. tested this therapy in a fully allogeneic rat heart transplantation model to prevent Cardiac Allograft Vasculopathy (CAV). Before transplantation into Lewis rats, which were immunosuppressed with cyclosporine, WF rat cardiac allografts were treated ex vivo with STAT‐1, AP‐1, or a buffer solution. Compared to the control, both decoy ODNs significantly reduced CAV severity and incidence. On day 1 post‐transplantation, laser‐assisted microdissection, RT‐PCR, and immunohistochemistry revealed a notable increase in CD40 levels in medial SMCs and coronary endothelial cells. Treatment with AP‐1 or STAT‐1 decoy ODNs nearly eliminated this increase. The AP‐1 decoy ODN lowered baseline CD40 expression in rat natural endothelial cells, while the STAT‐1 ODN suppressed TNF‐α/INF‐ɤ‐stimulated CD40 expression. During donor heart transplantation, decoy ODNs targeting STAT‐1 or AP‐1 inhibit CD40 upregulation in graft coronary arteries, helping to prevent CAV [21].

Xie et al. examine how AP‐1 ODNs inhibit collagen formation and AngII‐related proliferation in cardiac fibroblasts of newborn rats. After 24 h in serum‐free media, neonatal SD rat CFs were treated with AngII along with AP‐1 decoy ODNs or mutated ODNs at different doses. As the concentration of AP‐1 decoy ODN increased, CF absorbance decreased in the MTT test. Compared to the AngII group, 100 or 200 nmol/L AP‐1 decoy ODNs significantly lowered CF OD490. Hydroxyproline levels rose notably after AngII treatment compared to the control. Cells treated with either 100 or 200 nmol/L AP‐1 decoy ODNs showed much lower hydroxyproline levels than those treated with AngII. In addition to reducing hydroxyproline levels, AP‐1 decoy ODNs decreased S‐phase cells and increased G0/G1 cells. Collagen synthesis and AngII‐induced CF proliferation were not affected by AP‐1 decoy ODNs at 100 and 200 nmol/L. By altering cell cycle distribution, the AP‐1 decoy may reduce AngII‐induced proliferation and collagen production in rat CFs [57].

Xie et al. transfected CFs with decoy ODNs containing the AP‐1‐binding regions to reduce CF proliferation and MMP production by interacting with the transcription factor AP‐1. Cultured Sprague–Dawley rat cardiac fibroblasts were exposed to various doses of XXO, xanthine and AP‐1 decoy ODNs. MMP expression was evaluated after oxidative stress and transfection with AP‐1 decoy ODNs, using RT‐PCR and Western blot analysis. The dose‐dependent DNA‐binding activity of AP‐1 was significantly increased by XXO. In vitro, transfection with AP‐1 decoy ODNs decreased XXO‐induced MMP gene expression and CF proliferation. They concluded that AP‐1 plays a crucial role in MMP production and CF proliferation in response to oxidative stress. MMP production and CF growth may be inhibited by AP‐1 decoy ODNs [58].

Both SMAD‐independent and SMAD‐dependent pathways are utilized by the TGF‐β signalling pathway to promote tissue fibrosis and scarring. The antifibrotic benefits of TGF‐β inhibitors are diminished when only SMAD‐mediated signal transduction is blocked because this leads to an excessive inflammatory response. Yuan et al. described the design and in vitro and in vivo characterization of antifibrosis ODN 4 (AFODN4), a dual‐functional transcription activator that acts as both an AP‐1 and SMAD decoy ODN. AFODN4 has a high affinity for directly binding to SMAD and recombinant AP‐1. In L929 mice fibroblasts, AFODN4 significantly reduced the transcriptional and DNA‐binding activities of SMAD and AP‐1, as well as fibrotic mediators triggered by TGF‐β1 or ‐β2. When administered locally, AFODN4 notably decreased fibrosis associated with acute cutaneous wounds in mice. Since AFODN4 blocks SMAD both in vitro and in vivo, it effectively inhibits AP‐1‐induced production of pro‐inflammatory mediators. The findings indicate that the combined suppression of SMAD and AP‐1 signalling by AFODN4 presents a promising strategy for developing new antifibrotic therapies [59].

Marfan syndrome is characterized by increased levels of MMP in aortic smooth muscle cells (AoSMCs), which are associated with medial elastolysis and the formation of aortic root aneurysms. Arif et al. improved aortic elastolysis by reducing MMP expression using decoy ODNs that neutralize AP‐1. Administering AP‐1 decoy ODN significantly decreased both the migration and production of superoxide radical anions in mAoSMCs. Aortic grafts from donor Marfan mice were treated ex vivo with AP‐1 decoy ODN and later used as infrarenal aortic interposition grafts in mgR/mgR mice. Initial treatment of aortic grafts with AP‐1 decoy ODN resulted in decreased macrophage infiltration, MMP activity, and elastolysis. In mgR/mgR aortae, decoy ODN improved endothelial monolayer permeability, which was associated with reduced levels of the tight junction proteins occludin and ZO‐1. This enabled dODN to penetrate the tunica media. A new strategy for addressing the vascular features of Marfan syndrome involves targeting AP‐1 activation [60].

Transplant vasculopathy (TV) is the main obstacle to long‐term graft survival, characterized by myofibroblast activity, fibrosis, and the proliferation of smooth muscle cells (SMCs). The decoy ODN targeting the transcription factor AP‐1 may suppress the AV‐specific genes involved in neointima formation. Aortic allografts from DBA/2 mice were transplanted into the infrarenal aorta of C57BL/6 mice after exposure to either consensus, control buffer, or mutant control AP‐1 dODN. Daily injections of cyclosporine A at 10 mg/kg body weight were administered. The neointima showed a significant decrease in SMC α‐actin‐2 staining and macrophage marker expression. Using intraoperative AP‐1 dODN may help maintain graft function after transplantation [61].

TV is characterized by obstructions in affected vessels and is a major long‐term complication of cardiac transplantation. The activation of AP‐1 correlates with the phenotypic transition of SMCs from a contractile to a synthetic state, leading to increased proliferation and migration of these cells. Researchers suggested that administering an RNA hairpin AP‐1 dON via adeno‐associated virus (AAV) could significantly reduce the severity of TV in a mouse aortic allograft model. The infrarenal aorta of C57BL/6 mice was transplanted with aortic allografts from DBA/2 mice that had been ex vivo transduced using a modified AAV9‐SLR with a target peptide on the capsid surface. AP‐1 dON therapy significantly decreased the grafts' intima‐to‐media ratio by 41.5%. The treated aortic grafts showed downregulation of adhesion molecules, cytokines and reductions in MMP‐9‐positive cells, proliferative VSMCs and inflammatory cell infiltration. These findings demonstrate the potential, effectiveness and sensitivity of the anti‐AP‐1 RNA dON strategy in targeting allograft vasculopathy within an animal model. The extended distribution of nucleic acid therapies to the vascular wall is enabled by the AAV‐based approach [62].

Mutations in the fibrillin‐1 gene cause Marfan syndrome, a common hereditary connective tissue disorder. The disease features increased TF AP‐1 DNA binding activity, which elevates MMP activity. To prevent aortic elastolysis in a mouse model of Marfan syndrome, Remes et al. developed a new adeno‐associated virus (AAV)‐based method for sustained production of an AP‐1 antagonistic RNA hairpin dON in the aorta. The transduction led to the persistent presence of the therapeutic decoy ODN in smooth muscle and endothelial cells. Consequently, reactive oxygen species production, monocyte chemoattractant protein‐1 expression, and MMP expression and activity were all significantly reduced. The structural integrity of the elastin architecture was maintained, and monocyte graft penetration was decreased. The beneficial effect of AP‐1 antagonism on the pro‐inflammatory microenvironment in smooth muscle cells was confirmed by RNAseq analysis. This approach preserves aortic stability by continuously delivering nucleic acid‐based therapeutics and provides insight into the mechanism of elastolysis [63].

## 8 Discussion

AP‐1, an essential transcription factor, participates in several biological activities, including apoptosis, differentiation, survival, cell migration, proliferation, and transformation. AP‐1 has emerged as an important target in drug development, garnering considerable interest over the previous two decades, particularly in recent times. Evidence reviewed in this study indicates the significant potential of AP‐1 decoys as treatments for a range of disorders in which inflammatory pathways play a critical role in pathogenesis, including cardiovascular ailments and malignancies. Considering the significant therapeutic promise of this target and the considerable expenditures from both academic and industrial sectors, only one selective AP‐1 inhibitor is now prepared to commence human clinical trials. According to human and animal studies conducted using various decoy ODNs, it has been shown that decoys do not cause unwanted immunological reactions and overall do not cause any significant or specific side effects [43, 72, 73]. The use of decoy ODNs in therapeutic environments encounters obstacles, such as inadequate cellular absorption of unmodified ODNs and destruction by cellular nucleases.

As with any innovative treatment approach, decoy technology has some limits and obstacles that must be adequately addressed before clinical use. The primary obstacles relate to structural instability, cellular absorption efficiency, and an appropriate delivery method. The stability of the decoy has been strengthened by the use of circular ODN in a dumbbell form, resulting in greater stability and effectiveness. The enduring effects of ODNs are contingent upon their mode of administration [66]. Consequently, certain drug delivery techniques such as ultrasound‐targeted microbubble destruction using ODN‐coated microbubbles or adeno‐associated viral (AAV) vectors for tissue‐specific transduction and sustained expression in non‐dividing cells have been investigated. Notably, among these techniques, encouraging outcomes have been shown for in vivo drug administration using targeted nanoparticles. The simplicity of manufacture and substantial loading capacity have rendered nanoparticles indispensable alternatives to current delivery systems.

The enduring impact of decoys mostly depends on their stability and distribution efficiency. Local delivery is seen as the simplest and most efficient approach to addressing systemic administrative issues. Local delivery is feasible just for a limited number of conditions, such as those affecting the eyes, skin, and lungs [66], for the majority of treatment approaches, the optimal method is systemic delivery. Furthermore, decoy distribution may be classified as either passive‐targeting or active‐targeting delivery. Passive targeting is used for tumours and other highly permeable organs. Conversely, active targeting of nanoparticles is used for certain cell types or tissues. The latter is suitable for peripheral tissues, such as muscles, and tissues with limited permeability [74]. The use of TFDs, especially in conditions necessitating systemic administration, is mostly constrained by two challenges: the negative charge of these constructs, which hinders cellular uptake, and their vulnerability to nucleases, resulting in instability [73, 75]. Most mammalian cells can absorb enough TFDs; nevertheless, under physiological conditions, it is essential to consider active transport via delivery mechanisms and/or receptor‐mediated endocytosis to optimize cellular absorption and, therefore, the efficacy of TFDs [66].

Combination therapy such as NFKβ‐ and STAT3‐targeting ODNs, which have advanced to clinical trials, may provide significant advantages for two distinct reasons. Initially, because each drug operates via distinct molecular mechanisms, the likelihood of resistance is less in comparison to monotherapy. The dose of chemical medications may be reduced to a level that minimizes unwanted effects while maintaining antitumor efficacy with little alterations. Consequently, two therapeutic strategies may concurrently target essential cancer processes synergistically. Off‐target effects represent a significant concern in the use of decoy ODNs. The meticulous design of the decoy sequence is crucial to minimize non‐specific binding to other transcription factors. Robson et al. conducted research demonstrating that siRNA‐mediated inhibition of the PAX2 transcription factor induces apoptosis in bladder cancer cells. Nonetheless, transfection with decoy ODN significantly inhibited cell proliferation. The studies demonstrated that decoy ODN transfection had off‐target effects that suppressed cell proliferation [76]. In several other studies, researchers may not have evaluated the off‐target impact of decoy ODNs on the expression of other genes. Consequently, it is essential to exercise care in assessing the specificity of decoy ODN sequences prior to contemplating their use as a prospective therapeutic agent.

An other critical issues in this field is context‐dependent and dimer‐specific actions of AP‐1 decoys, which should be cerfully considered. Because AP‐1 dimers can differ in DNA‐binding affinity, cofactor recruitment, and cellular responsiveness, decoy activity could be not uniform and may influence multiple downstream pathways depending on the cellular state and AP‐1 composition. The c‐Jun and c‐fos proto‐oncogenes produce proteins that create a complex responsible for regulating transcription from promoters that contain AP‐1 activation elements. c‐Jun exhibits specific DNA binding activity, whereas c‐Fos shows homology to the presumed DNA binding domain of c‐Jun. After in vitro translation, c‐Jun attaches as a homodimer to the AP‐1 DNA site, while c‐Fos does not dimerize and shows no significant affinity for the AP‐1 element. When cotranslated, c‐Jun and c‐Fos proteins bind to the AP‐1 DNA site 25 times more effectively as a heterodimer compared to the c‐Jun homodimer [77]. Although Jun proteins are capable of forming both homo‐ and heterodimers with various proteins, Fos proteins are restricted to heterodimerization solely with Jun proteins. Additionally, Fos/Jun heterodimers exhibit greater stability compared to Jun homodimers [78, 79]. The activated AP‐1 is capable of binding to a specific DNA sequence, 5′‐TGAG/CTCA‐3′, found within the promoter or enhancer region, thus regulating the transcription of downstream target genes [80]. The DNA‐binding activity of this molecule can be augmented by 12‐O‐tetradecanoylphorbol 13‐acetate (TPA). Consequently, its DNA binding motif is referred to as the TPA‐response element (TRE) [81]. Furthermore, AP‐1, which consists of a Jun/ATF heterodimer, exhibits a greater affinity for a different DNA sequence, 5′‐TGAGCGTCA‐3′, that responds to cyclic AMP and is designated as the cyclic AMP responsive element (CRE) [7]. Upon activation, AP‐1 modulates the expression of downstream genes that are involved in various processes, including cell growth, apoptosis, angiogenesis, and drug resistance [82, 83]. As a mechanism through which it influences cell proliferation, AP‐1 can additionally co‐occupy chromatin alongside YAP/TAZ, the nuclear effectors of the Hippo pathway, thereby regulating downstream genes that govern S‐phase entry and mitosis. Furthermore, the relationship between AP‐1 and YAP/TAZ complexes may also facilitate skin tumorigenesis [82, 84]. Some members of the AP‐1 family have been identified as having functions in the invasion of tumour cells. Various Jun and Fos family members have been noted to engage with SMAD proteins that are crucial for the epithelial to mesenchymal transition (EMT) and the ensuing invasion associated with breast cancer. For instance, the AP‐1 members c‐Jun and JunB are known to interact with Smad3, while Fra‐1 can form a complex with Smad2/3 following TGFβ stimulation; however, c‐Fos is not necessary for this interaction [85, 86]. JunB exhibits oncogenic properties; when stimulated by TGF‐β, it can further influence downstream genes that are associated with tumour invasion and progression [87]. c‐Jun, JunB, c‐Fos and Fra‐1 all participate in the regulation of the cell cycle via cyclin A [88]. Additionally, the Fos family significantly contributes to tumour progression. Fra‐1 has been found to have a positive correlation with cancer malignancy, proliferation, and invasion [79, 89]. Hence, AP‐1 perturbation influences wide gene networks (not just canonical targets) underscoring its pleiotropic nature and the limitations of earlier small gene‐panel analyses. Given the growing availability of transcriptomic and genome‐wide data, wider downstream effects of genes (pleiotropy) have been highlighted and the limitations of earlier gene‐panel studies have been minimized.

## 9 Conclusion

AP‐1, a vital transcription factor, is involved in numerous cellular functions, including survival, differentiation, cell migration, proliferation, apoptosis, and transformation. AP‐1 has become a prominent target in drug discovery, attracting significant attention over the past two decades, especially recently. Evidence shows the great, promising role of AP‐1 decoys as therapeutics for various diseases, especially cardiovascular diseases and cancers. Given the substantial therapeutic potential of this target and the large investments from both academic and industry sectors, only one selective AP‐1 inhibitor is currently ready to enter human clinical trials. Using decoy ODNs in clinical settings faces challenges, including poor cellular uptake of unmodified ODNs and degradation by cellular nucleases. Recent studies reveal that various nanocarriers effectively address the issue of low cellular uptake. Moreover, it was shown that modifications of AP‐1 (circular and hairpin as well as phosphorothioate backbone structures) decoys made them more stable and effective. Future clinical studies could shed more light on the effects of AP‐1 decoys in the human body.

## Author Contributions

**Vasily N. Sukhorukov:** writing – original draft. **Maryam Mahjoubin‐Tehran:** writing – original draft. **Amirhossein Sahebkar:** conceptualization, writing – review and editing, supervision. **Prashant Kesharwani:** conceptualization, writing – review and editing, supervision. **Samaneh Rezaei:** writing – original draft. **Amin Jalili:** writing – original draft.

## Funding

This work was supported by the Russian Science Foundation (Grant # 25‐15‐00269).

## Conflicts of Interest

The authors declare no conflicts of interest.

## Data Availability

The authors have nothing to report.
